# Adding arterial nitrogen pressure to single‐measurement monitoring data enables diagnostic lung modeling by deep learning

**DOI:** 10.14814/phy2.70647

**Published:** 2026-02-16

**Authors:** Peter H. Scott, Christopher M. Anstey, Thomas J. Morgan

**Affiliations:** ^1^ Intensive Care Department, Mater Health Services Brisbane Queensland Australia; ^2^ University of Queensland Brisbane Queensland Australia; ^3^ Griffith University Gold Coast Queensland Australia; ^4^ Mater Research Brisbane Queensland Australia

**Keywords:** blood gases, deep learning, lung model, nitrogen pressure, shunt, V/Q mismatch

## Abstract

We investigated whether including arterial pressure of nitrogen (PaN_2_) in a deep‐learning analysis of single measurements of arterial blood gases, cardiac output, and indirect calorimetry enables individualized quantification of West's ventilation/perfusion (V/Q) lung model. West's key parameters are shunt (% cardiac output supplying lung units with V/Q = 0), logSD (log standard deviation of unit V/Q ratios), and meanV/Q (mean unit V/Q ratio). By processing randomized combinations of shunt, logSD, meanV/Q, indirect calorimetry, and cardiac output data in a Python computerization of West's model, 2,010,000 blood gases including PaN_2_ combined with their input variables completed a simulated monitoring dataset covering broad ranges of oxygenation and acid–base equilibria. Deep‐learning applications trained on these data successfully predicted withheld values of shunt, logSD, and meanV/Q from a separate test dataset of 43,915 samples. Linear regression of predicted versus true values produced *R* ^2^ ≥ 0.99 with slopes 0.98–1.00. Kernel density estimates confirmed close agreement. Sensitivity analyses demonstrated high dependence upon PaN_2_. Deep‐learning analysis of single measurements of arterial blood gases, which include PaN_2_, when combined with cardiac output and indirect calorimetry data, can quantify individual lung function with high fidelity in terms of key parameters of West's V/Q model.

## 1 INTRODUCTION

### 1.1 Some background

Modeling pulmonary gas exchange is challenging, not least because the lung is a complex structure with an estimated mean alveolar count of 480 × 10^6^ (Ochs et al., 2004). Riley and Cournand's “three‐compartment” model was a landmark approach, albeit a major simplification (Riley & Cournand, 1951). In their model, gas exchange is confined to a single “ideal” compartment with matched ventilation and perfusion (V/Q = 1) and accompanied by a zero ventilation or “shunt” compartment (V/Q = 0) plus a zero perfusion or “dead space” compartment (V/Q = ∞).

“Venous admixture,” derived from this model (Nirmalan et al., 2001), denotes the calculated quantity of mixed venous blood perfusing the shunt compartment, which, when mixed with blood from the ideal compartment, reproduces the blood gas characteristics of arterial blood. Expressed as a percentage of cardiac output, venous admixture is still in clinical use, as are other model—derived parameters such as the A‐a gradient (Zimmerman et al., 2006) and dead space as a proportion of total ventilation (Robertson, 2015).

All display signal variability (Kathirgamanathan et al., 2009) and oversimplify pulmonary pathophysiology. For example, venous admixture consists of varying contributions from true shunt (V/Q = 0) as well as from lung units with V/Q ratios <1, whereas the dead space compartment comprises “true” dead space plus contributions from units with V/Q ratios >1 (see Supporting Information File 1: Sample Solutions; Sections 1 and 2).

West retained the shunt compartment but added 50 gas‐exchanging compartments with V/Q ratios distributed lognormally (West, 1969). With Wagner, he introduced the multiple inert gas elimination technique (MIGET) (Wagner, 2008) to facilitate individual diagnosis based on this model. MIGET entails measurements of the uptake and retention of six inert gases of varying solubility, plus an optimization or “smoothing” algorithm to facilitate data fitting.

Due to its technical challenges, “MIGET alternatives” like the automatic lung parameter estimator (ALPE) have been proposed. ALPE has shown promise experimentally (Rees et al., 2006, 2010) and in clinical research (Karbing et al., 2020). Although less complex, ALPE requires an extended series of measurements, while its quantification of “low” and “high” V/Q mismatch as partial pressure differentials across notional blood/alveolar gas partitions is less intuitive.

### 1.2 The work of our group

With the advent of machine learning, our group has produced evidence that diagnostic West model adaptations can be applied without MIGET's technical complexity. Just as venous admixture can be calculated using data from blood gas analysis, cardiac output measurement, and indirect calorimetry, we have demonstrated in silico that machine learning applications trained on the same inputs can recover the key West model parameters necessary for individual diagnosis (Morgan, Langley, et al., 2023; Morgan, Scott, et al., 2023).

In the “Two‐FiO_2_” approach, data generated at two settings of FiO_2_ (Morgan, Barrett, & Anstey, 2023) were sufficient when analyzed by machine learning to define the three key parameters of the West V/Q lung model (West, 1969, 1977). These are shunt (the percentage of cardiac output supplying lung units where V/Q = 0), logSD (the log of the standard deviation of the distribution of unit V/Q ratios), and meanV/Q (the mean V/Q ratio of lung units). The subsequent “Single–FiO_2_” modification (Morgan, Scott, et al., 2023) quantified the shunt versus “low V/Q” contributions to venous admixture. This method successfully removed the second FiO_2_ step by adding volumetric capnometry estimates of the mean alveolar partial pressure of carbon dioxide (mPACO_2_) to the input data (Suarez‐Sipmann et al., 2014). Volumetric capnometry provides data including excretion of CO_2_, alveolar ventilation, dead space and mPACO_2_.

#### 1.2.1 A potential caveat

Avoiding a second FiO_2_ phase eliminates signal distortion due to altered atelectasis or varying hypoxic pulmonary vasoconstriction. However, including volumetric capnometry data for this purpose introduces a potential drawback since its measurements can be impacted by “true” alveolar dead space (V_Dz1_), defined as the fraction of alveolar ventilation distributed to non‐perfused alveoli (V/Q = ∞) and analogous to lung units collectively described by West as forming “Zone 1” (West et al., 1964).

Although V_Dz1_ is normally minuscule and difficult to quantify (Robertson, 2015), its value can increase significantly when there is widespread pulmonary capillary hypoperfusion, for example, with severe hypotension, thromboembolism, or diffuse capillary microthrombi as described in COVID‐19 pneumonitis (Gattinoni et al., 2021). The consequent alveolar dilution with unmodified inspiratory gas reduces mPACO_2_. Deep‐learning predictions of shunt will then be underestimated, reducing shunt's contribution to venous admixture while exaggerating the notional influence of low V/Q lung units (Morgan, Scott, et al., 2023).

#### 1.2.2 The current project

Substituting arterial nitrogen pressure (PaN_2_) for mPACO_2_ would circumvent this drawback, since pulmonary nitrogen transfer is limited to perfused and ventilated alveoli and unaffected by increases in V_Dz1_. Evidence for trialing this strategy includes Canfield and Rahn's observations linking perfusion of low V/Q units to the alveolar‐arterial PN_2_ gradient (Canfield & Rahn, 1957) and the demonstration of a close relationship between the alveolar‐arterial PN_2_ gradient and perfusion of lung regions where V/Q < 0.9, facilitating shunt estimates from venous admixture calculations (Radermacher et al., 1988; Radermacher & Falke, 1987).

We therefore tested the following hypothesis in silico:

“Deep‐learning analysis of data collected at a single FiO_2_ from single measurements of indirect calorimetry, cardiac output, and blood gases which include PaN_2_ can quantify pulmonary gas exchange in terms of the three defining West model parameters: shunt, logSD, and meanV/Q.”

## 2 METHODS

**FIGURE 1:**
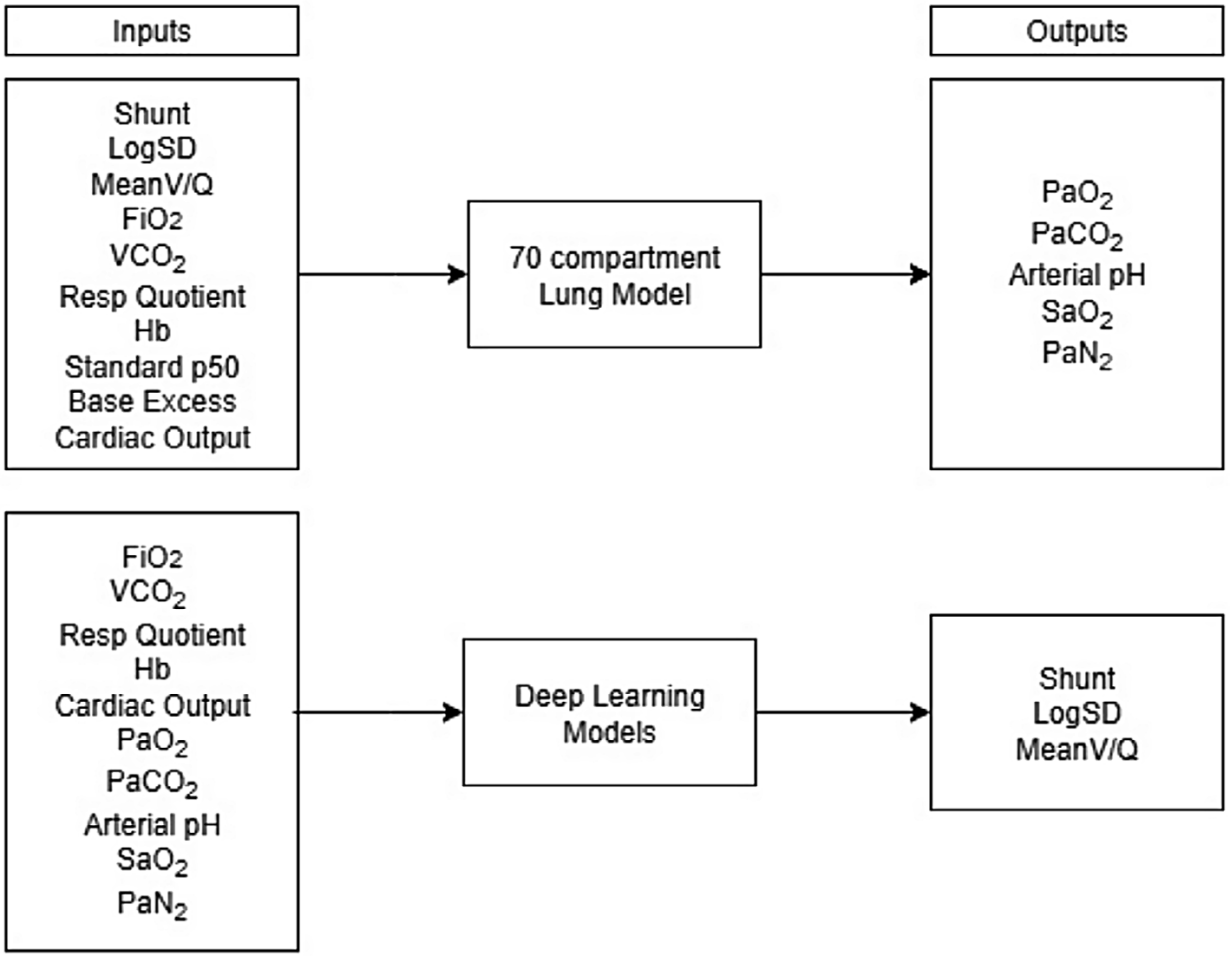
Schematic of the flow of investigation. Upper boxes display inputs for the multicompartmental lung model that generated arterial blood gas values to build the training dataset. Lower boxes display inputs from the training dataset used to train the deep learning models to estimate the difficult‐to‐measure parameters shunt, logSD, and meanVQ. After training, the deep learning models processed case sample inputs from the test database, yielding the required outputs. FiO_2_, inspired oxygen fraction; Hb, hemoglobin concentration; PaCO_2_, carbon dioxide partial pressure in arterial blood; PaN_2_, arterial pressure of nitrogen; PaO_2_, oxygen partial pressure in arterial blood; SaO_2_, hemoglobin–oxygen saturation, VCO_2_, CO_2_ production.

To test the hypothesis, we used a multicompartmental lung model to generate a dataset of two million simulated case samples from random inputs. With this dataset, we trained deep learning models to recover the defining parameters of the lung model from readily measurable features as inputs. We then evaluated the trained deep learning models on a separately generated test dataset (Figure 1). We also explored the sensitivity of each input feature to measurement errors.

A 70‐compartment computerized version of the West lung model (Scott & Morgan, 2024) was programmed using Python (version 3.11.8) (Python Software Foundation, n.d.) to simulate monitoring data covering a broad range of oxygenation and acid–base equilibria. Values of Q and V for each compartment were defined by three key model parameters, consisting of shunt, logSD, and meanV/Q. Other variables entered into the simulation were FiO_2_, CO_2_ production (VCO_2_), respiratory quotient R (the ratio of VCO_2_ to oxygen consumption), hemoglobin concentration (Hb), base excess (Siggaard‐Andersen, 1963), standard P50 (Morgan, 1999; Severinghaus, 1979), and cardiac output (Q_T_). Also entered as constants were barometric pressure = 760 mm Hg, temperature = 37°C, and inspired PCO_2_ = 0 mm Hg.

Values of input variables were selected randomly from their assigned physiological ranges (Table 1), all of which were linear apart from the logarithmic meanV/Q range. Arterial blood gases, which included PaN_2_ values, were calculated for each randomized combination of inputs, with results pre‐defined as “extreme” (Table 2) removed during the process. A training dataset of 2,010,000 samples was then created linking each set of blood gases to its corresponding input variables. A separate test dataset of 50,000 samples was generated by the same method, of which 43,915 remained after further filtering (Table 2) to better emulate clinical scenarios.

**TABLE 1:** Monitoring inputs with ranges used by the lung model to generate blood gases and respiratory profiles.

Variable	Range
Shunt (% of pulmonary blood flow)	0–50
LogSD	0.40–2.0
MeanV/Q	0.30–3.0
FiO_2_	0.20–1.0
Rate of CO_2_ production (mL/min)	100–450
Respiratory quotient	0.7–1.0
Hemoglobin (g/dL)	3.0–21.0
Standard P50 (mm Hg)	20.7–33.7
Base excess (mEq/L)	−25 to +20
Cardiac output (L/min)	4.0–8.0

Abbreviation: FiO_2_, inspired oxygen fraction.

**TABLE 2:** Model‐generated blood gas inclusion ranges.

Variable	Range	Reduced range for test dataset
pH	6.8–8.0	6.8–7.8
PaO_2_ (mm Hg)	Greater than 0	Greater than 40
PaCO_2_ (mm Hg)	Greater than 0	10–200
PvO_2_ (mm Hg)	Greater than 0	
PvCO_2_ (mm Hg)	Greater than 0	
Venous admixture (%)	<90	
Alveolar dead space (%)	0–90	
Alveolar ventilation (L/min)	1.5–40	

*Note*: The third column displays additional restrictions for inclusion in the test dataset.

Abbreviations: PaCO_2_, carbon dioxide partial pressure in arterial blood; PaO_2_, oxygen partial pressure in arterial blood; PvO_2_, oxygen partial pressure in mixed venous blood; PvCO_2_, carbon dioxide partial pressure in mixed venous blood.

The training dataset was used to train deep‐learning models with architectures previously aimed at shunt prediction (Morgan, Scott, et al., 2023) to recover shunt, logSD, and meanV/Q values from single inputs of ten monitoring data elements. These were FiO_2_, VCO_2_, R, Q_T_, PaN_2_, plus five routine arterial blood gas measurements consisting of Hb, pH, partial pressure of O_2_ (PaO_2_), partial pressure of CO_2_ (PaCO_2_), and hemoglobin–oxygen saturation (SaO_2_). Recovered shunt, logSD, and meanV/Q values were then compared with corresponding true values.

### 2.1 Statistical analysis

For each of the three target parameters, there was a dataset consisting of binary pairs of actual and estimated values. Univariate regression modeling was performed on each data pair, with the predicted variable assigned as the dependent variable and its “gold standard” value as the independent variable. Regression slopes (*β*) with 95% confidence intervals and resulting *p*‐values were reported. Overall model fit was documented using the coefficient of determination (*R* ^2^) value. The level of significance was set at *α* < 0.05 throughout. STATA™ (STATACorp) version 17.0 (StataCorp, 2023) was used for all analyses.

Kernel density estimate (KDE) distributions were generated to present results graphically. These incorporated Gaussian kernels, with smoothing bandwidths determined by the “Scott Rule” (Scott, 1992). All figures were generated in Python using Matplotlib (version 3.3.2) (Hunter, 2007) and Seaborn (version 0.11.0) (Waskom, 2021).

### 2.2 Sensitivity analysis

Python SHAP version 0.44.1 (Lundberg, n.d.) yielded SHAP (also known as Shapely) values to quantify the sensitivity of predictions to input features. In this approach, input features for a given sample are varied and consequent changes to target parameter prediction are evaluated using cooperative game theory (Lundberg & Lee, 2017). Induced variations in the target parameter are quantified as a SHAP value for each input feature averaged over multiple samples. Thus, the relative contributions of the respective input features to the deep‐learning model can be ranked. SHAP values are additive and represent the amount by which the input feature moved the result from an average expected result to the observed result. From the test dataset, in accordance with sampling theory, 5000 examples were randomly selected for calibration of the SHAP model, and a further 500 examples were randomly selected for the sensitivity analysis. These representative subsets were mutually exclusive (Cochran, 1977; Krejcie & Morgan, 1970; Moore et al., 2012).

### 2.3 Deep‐learning models

The “Keras” library (version 2.10) (Chollet, 2015, 2021) was used to construct the deep‐learning models. The architecture of each model involved an input layer of 10 features, six densely connected intermediate layers with 128 units/layer, and a final layer with one unit being the output. Thus, each model had 84,097 trainable parameters. The activation function was ReLU, the optimizer was RMSprop, and the loss function was mean squared error. Usual practices of dataset shuffling, normalization of input features, and avoidance of overfitting were followed. Three models were trained to predict shunt, logSD, and meanV/Q, respectively, using 200 epochs of the training dataset. Background was published previously (Morgan, Scott, et al., 2023), and further details are included in Supporting Information File 1.

## 3 RESULTS

**FIGURE 2:**
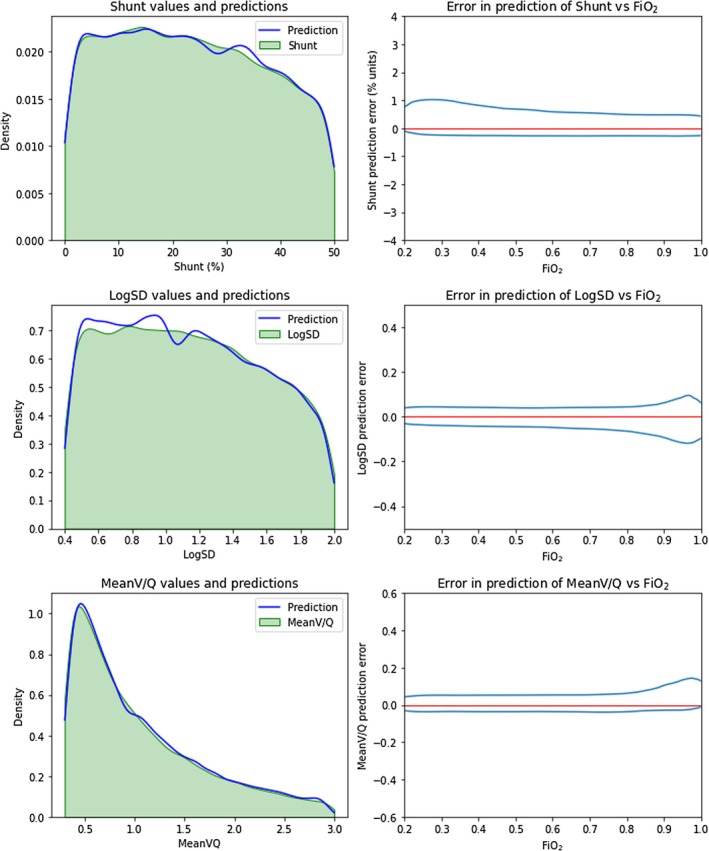
*Left‐hand panels*: kernel density estimates of distributions for shunt, logSD, and meanV/Q in the test dataset, comparing true values and values predicted by trained deep‐learning models. *Right‐hand panels*: Bivariate kernel density estimates of error in the prediction of shunt, logSD, and meanV/Q, respectively, versus FiO_2_. Contours contain 95% of the values. The red line represents zero error. Notably, the error band widens at FiO_2_ > 0.85 for the prediction of logSD and meanV/Q. Conversely, the band narrows for shunt, reflecting the diminution of the contribution of V/Q mismatch to venous admixture at high FiO_2_. *n* = 43,915.

Table 3 sets out comparisons of true versus predicted values. Coefficients of determination (*R* ^2^) for shunt predictions were essentially unity and were close to unity for logSD and meanV/Q predictions, consistent with a strong correlation. Figure 2 displays test sample KDE distributions of true shunt, logSD, and meanV/Q values with superimposed distributions of predicted values, along with plots of prediction error versus FiO_2_ for each target parameter. Strong correlation is again evident, reduced to some extent at low FiO_2_ for shunt and at high FiO_2_ for logSD and meanV/Q.

**TABLE 3:** Univariate linear regression for predicted parameters versus true values for shunt, logSD, and meanV/Q (*n* = 43,915).

	*β*	95% CI	*p* value	*R* ^2^
Shunt	+1.001	+1.001 to +1.002	<0.001	0.999
LogSD	+0.980	+0.979 to +0.981	<0.001	0.986
MeanV/Q	+0.992	+0.991 to +0.993	<0.001	0.996

*Note*: In each case, the dependent variable was the predicted value. Results are reported to three decimal places.

Abbreviations: *β*, regression coefficient value; CI, confidence interval; *R* ^2^, coefficient of determination.

**FIGURE 3:**
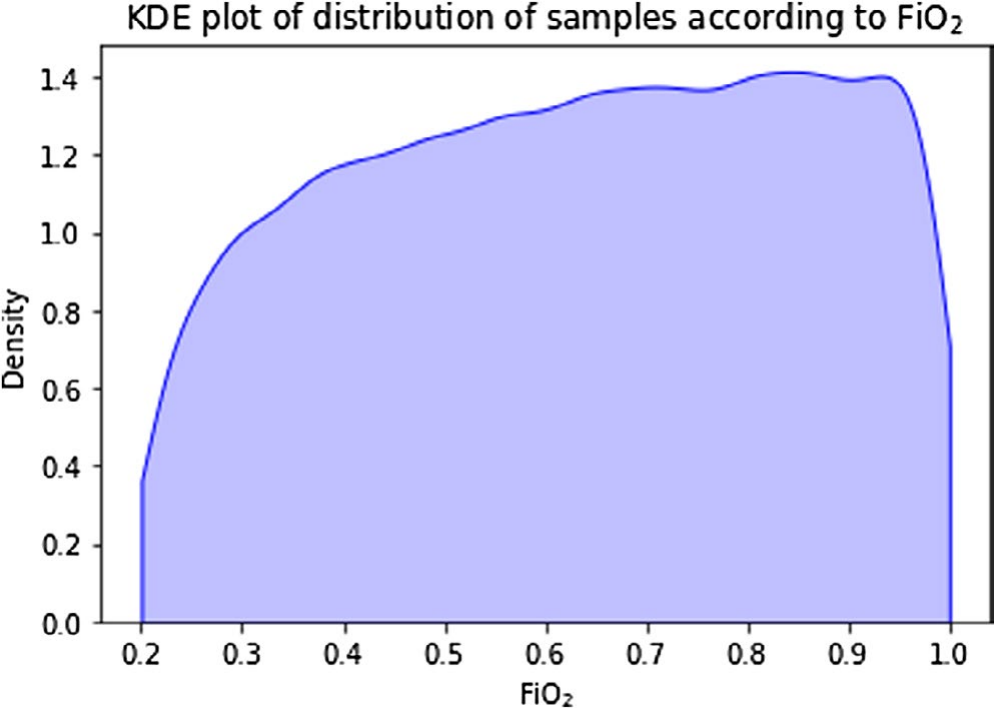
Kernel density estimate of the distribution of FiO_2_ in the test dataset. *n* = 43,915.

The distinctive shape of the meanV/Q KDE distribution (Figure 2) is consistent with its logarithmic source, while the apparent attenuation of shunt and logSD KDE distributions at higher values reflects a greater incidence of rejected outputs pre‐defined as “extreme” (Table 2). Figure 3 displays the KDE distribution of test samples according to FiO_2_. Again, the effects of output filtering are evident despite even input selections, with “extreme” results encountered more frequently at low FiO_2_ with higher settings of shunt and logSD. Figures S1–S3 in the Supporting Information File 1 contain further plots again confirming a strong correlation between deep‐learning predicted versus true values of the three key model parameters.

**FIGURE 4:**
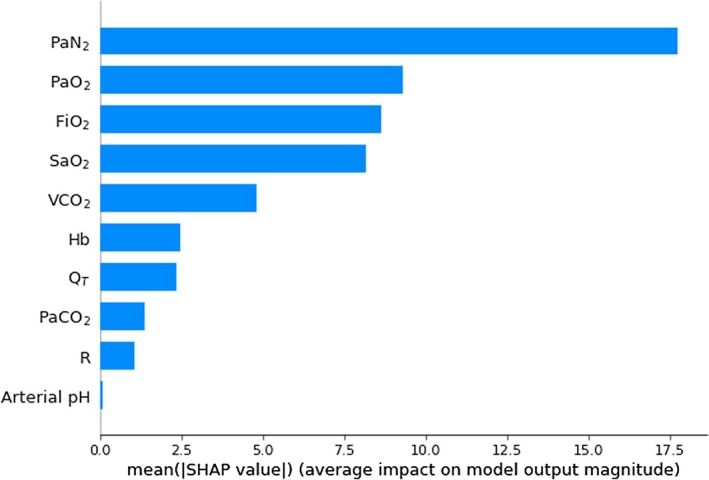
Histogram of the mean absolute SHAP values for 10 input features used by deep‐learning models in the prediction of shunt (*n* = 500), ranked from most important to least. FiO_2_, inspired oxygen fraction; Hb, hemoglobin concentration; Q_T_, cardiac output; PaCO_2_, carbon dioxide partial pressure in arterial blood; PaN_2_, arterial pressure of nitrogen; PaO_2_, oxygen partial pressure in arterial blood; R, respiratory quotient; SaO_2_, hemoglobin–oxygen saturation, VCO_2_, CO_2_ production.

**FIGURE 5:**
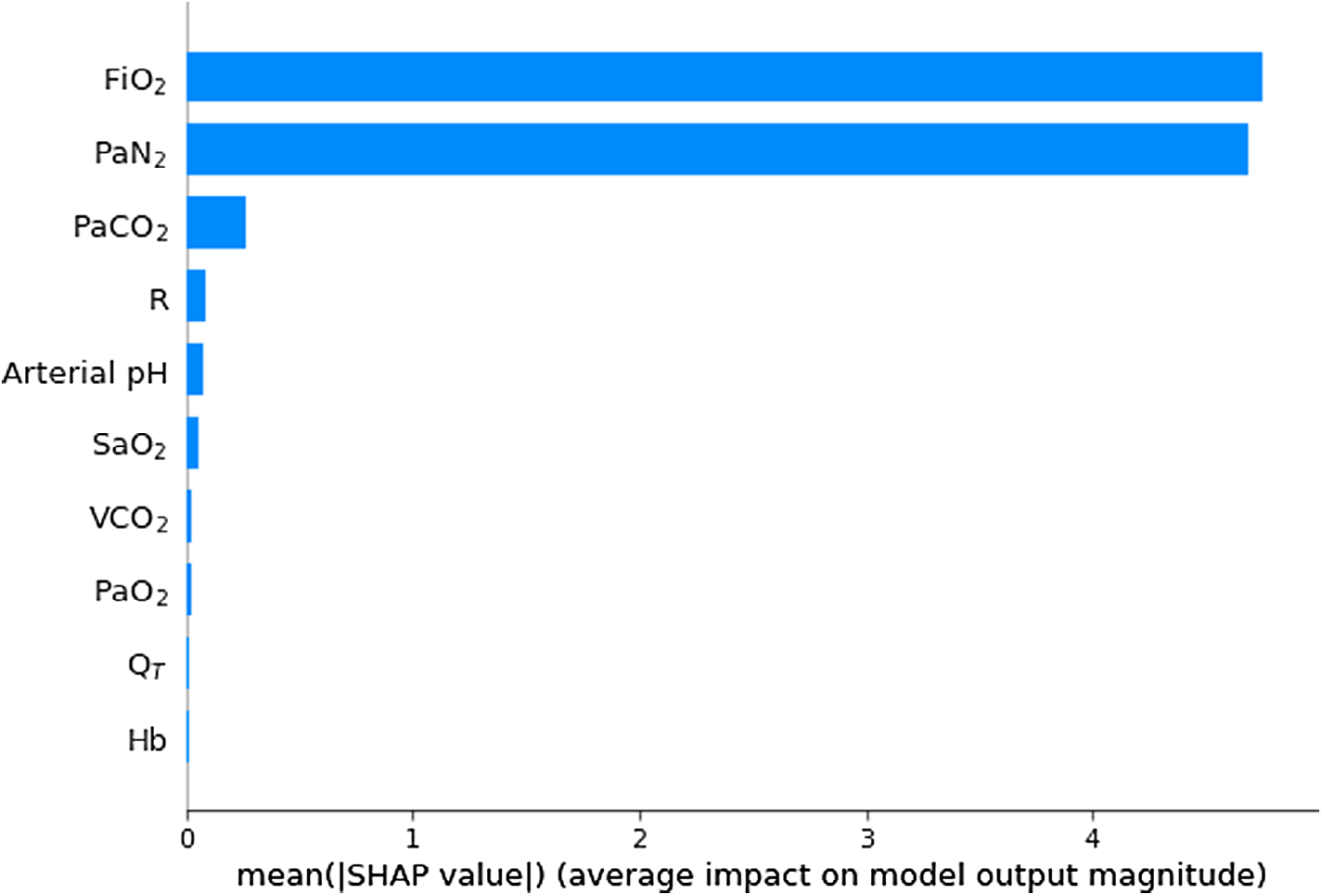
Histogram of the mean of absolute SHAP values for 10 input features used by deep‐learning models in the prediction of logSD (*n* = 500), ranked from most important to least. FiO_2_, inspired oxygen fraction; Hb, hemoglobin concentration; Q_T_, cardiac output; PaCO_2_, carbon dioxide partial pressure in arterial blood; PaN_2_, arterial pressure of nitrogen; PaO_2_, oxygen partial pressure in arterial blood; R, respiratory quotient; SaO_2_, hemoglobin–oxygen saturation, VCO_2_, CO_2_ production.

**FIGURE 6:**
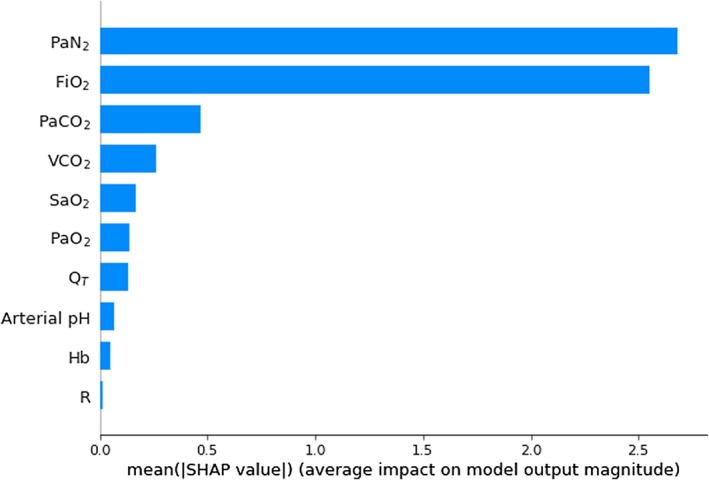
Histogram of the mean of absolute SHAP values for 10 input features used by deep‐learning models in the prediction of meanV/Q (*n* = 500), ranked from most important to least. FiO_2_, inspired oxygen fraction; Hb, hemoglobin concentration; Q_T_, cardiac output; PaCO_2_, carbon dioxide partial pressure in arterial blood; PaN_2_, arterial pressure of nitrogen; PaO_2_, oxygen partial pressure in arterial blood; R, respiratory quotient; SaO_2_, hemoglobin–oxygen saturation, VCO_2_, CO_2_ production.

Figures 4, 5, 6 are histograms of the mean absolute SHAP values for input features ranked by their contributions to the output. Figures S12–S14 in the Supporting Information File 1 illustrate further detail including variations in individual SHAP values and the relationship of effect direction with the magnitude of input feature. These histograms indicate that PaN_2_ sensitivity is prominent in deep‐learning predictions of all three key parameters.

## 4 DISCUSSION

This in silico simulation builds on previous findings (Morgan, Langley, et al., 2023) that shunt, logSD, and meanV/Q values—three key parameters of the West model of pulmonary gas exchange (West, 1969, 1977)—can be recovered in simulated patient cases by deep‐learning analysis of monitoring data readily measured at the bedside, and that accurate shunt predictions are possible without FiO_2_ manipulation when additional mPACO_2_ values, measurable by volumetric capnometry, are supplied. (Morgan, Scott, et al., 2023). The key takeaway from the current simulation is that accurate diagnostic “Single‐FiO_2_” modeling would be maintained if PaN_2_ values were to replace mPACO_2_ inputs.

As outlined in the Introduction, this substitution would further enhance the signal stability of “Single‐FiO_2_” modeling by eliminating distortions in conditions predisposed to increased V_Dz1_. It should be noted that reliable estimates of logSD and meanV/Q values were also recovered using PaN_2_ inputs. Although not investigated (Morgan, Scott, et al., 2023), these parameters should be similarly recoverable using mPACO_2_, but with their values susceptible to increased V_Dz1_.

All three model‐defining parameters are relevant when diagnosing pulmonary pathophysiology based on the West model. For example, the logSD parameter is a measure of dispersion that quantifies V/Q heterogeneity, either as a standalone metric or as a potential discriminator if used in conjunction with severity scoring systems such as those for acute lung injury and acute respiratory distress syndrome (ARDS) (Kangelaris et al., 2014; Wei et al., 2025).

From this perspective, the current RESP score, designed to predict survival in patients with ARDS managed with extracorporeal membrane oxygenation (Schmidt et al., 2014; Tonna et al., 2021) has had limited success in ARDS due to COVID‐19 (Pratt et al., 2023). A likely factor promoting RESP inaccuracy in COVID‐19 patients is the prominence of V/Q heterogeneity as a cause of oxygenation deficits (Gattinoni et al., 2021). With the West diagnostic model, this aberration would be flagged immediately by increased logSD estimates combined with relatively low shunt predictions. Since the same pathophysiology also promotes V_Dz1_ increases, a switch to PaN_2_ inputs would further enhance the diagnostic utility of Single‐FiO_2_ estimates.

In the current simulation, shunt predictions were most reliable at FiO_2_ ≥ 0.5, whereas logSD and meanV/Q predictions showed increasing scatter from approximately FiO_2_ ≥ 0.85 (Figure 2). The clustering of logSD and meanV/Q estimate outliers at high FiO_2_ suggests a diminution of discriminatory information, in parallel with the declining influence of V/Q mismatch on oxygenation as FiO_2_ increases, culminating in its complete negation at FiO_2_ = 1.0 (Scott & Morgan, 2024).

Paradoxically, the same phenomenon would tend to facilitate shunt predictions at high FiO_2_. A similar mechanism drives the convergence of venous admixture and shunt as the FiO_2_ approaches 1.0. This is why “low V/Q” contributions to oxygenation deficits are expressed as “venous admixture minus shunt” (Scott & Morgan, 2024) do not measure V/Q heterogeneity itself. For that, we need logSD (see also Supporting Information File 1: Sample Solutions; Section 1).

### 4.1 Novel estimates of V_Dz1_

To estimate V_Dz1_, differences could be exploited between mPACO_2_ values derived by “forward” West model calculations from PaN_2_‐based diagnostic outputs versus mPACO_2_ values quantified directly by volumetric capnometry, since only the latter respond to expired alveolar gas from Zone 1 areas. The gap between the two estimates could thus serve as an indirect index of V_Dz1_. In support of this concept, Figure S9 in the Supporting Information File 1 demonstrates the accuracy of PaN_2_‐based “forward” model estimates of mPACO_2_. More direct V_Dz1_ quantification would also be feasible. The lung model yields a value for alveolar ventilation which is the sum of expired volumes from all compartments. If inspired PCO_2_ = zero, the extra volume required to dilute the model‐derived mPACO_2_ to the volume capnometry‐measured mPACO_2_ represents V_Dz1_. These potential applications of the West diagnostic model are discussed further in Supporting Information File 1. A worked example of direct V_Dz1_ quantification is demonstrated in Supporting Information File 1: Sample Solutions; Section 2.

### 4.2 Sensitivity analyses

Determining the extent to which PaN_2_ measurements and other input features affect deep‐learning model accuracy can be challenging. Our study used SHAP values to quantify the contribution of respective input features to predictions of target parameters (Figures 4, 5, 6). Although this method quantifies the impact of measurement error of individual features, it does not account for interdependence between features.

SHAP‐based assessments demonstrated the high importance of PaN_2_ and FiO_2_ in the predictions of shunt, logSD, and meanV/Q. Their relatively low dependence on VCO_2_, R, and Q_T_ is reassuring, as these parameters are measured less consistently at the bedside, raising the possibility of “best estimates” being substituted when necessary.

### 4.3 Caveats and limitations

As a general criticism, our method, with its requirements of indirect calorimetry and cardiac output data, has limited potential application outside critical care.

As for the present simulation, rapid PaN_2_ measurements at the point of care are not currently available. To date, blood PN_2_ measurements have been manometric (Muth et al., 1994; Radermacher et al., 1988, 1990) or by gas chromatography (Corbet et al., 1974; Markello et al., 1972) and require a Van Slyke apparatus, whereas bedside diagnosis would necessitate an automated technique suited to commercial blood gas analyzers. The chemical inertness of nitrogen compared with oxygen and acidity adds to the difficulty. This is compounded by the need for accuracy over a relatively large range (e.g., from <20 mm Hg to >500 mm Hg), as illustrated by the impact of small PaN_2_ measurement errors in the present study. We hope our study encourages research into methods to overcome these obstacles and allow routine “bedside” PaN_2_ measurements.

Long equilibration periods may be necessary to ensure a stable N_2_ balance prior to measurements (Groom et al., 1967). Although the optimal equilibration interval is undetermined, meaningful PaN_2_ data were produced in adults when collection followed exposure to a constant FiO_2_ for a minimum of 1 hour (Radermacher et al., 1988).

High coefficient of determination values are consistent with our hypothesis that accurate recovery of the three key defining West model parameters is possible. When fitting the model to “real patient” clinical data, precision would be assessable by comparing West lung model forward calculations of blood gas values (using deep learning‐derived shunt, logSD, and meanV/Q), with “real” blood gases as gold standards.

We modeled symmetrical lognormal distributions of V/Q ratios, whereas MIGET analyses of real patient data have yielded asymmetric results (Melot, 1994). Of note, we did not set out to reproduce MIGET reports, the fidelity of which can also be questioned (see below), but rather to provide a simpler method of quantifying the degree of V/Q mismatch, the partitioning of venous admixture into shunt versus V/Q mismatch, and estimation of “true” alveolar dead space. To this end, a mathematical model must be fitted to case data, so that simplification will be required as with any mathematical model. The three‐compartment model of Riley and Cournand has been of clinical value although greatly simplified. Even the MIGET approach uses an optimization algorithm, albeit based upon six different gases, to find a model of best fit for measured data. Though detailed, it is nevertheless a simplification of the true situation.

Furthermore, the West model and MIGET approach both model V in the lognormal V/Q distribution as expiratory alveolar gas volumes (West & Wagner, 1977). We have shown that if inspiratory volumes are substituted within the same lognormal constraints (which is perhaps more intuitive), volume distributions of expired alveolar gas become asymmetric in less “healthy” lungs. This can manifest as a “hump” for low V/Q lung units, or even as “negative” expired volumes at higher FiO_2_ and logSD settings, which could trigger redistribution of inspired alveolar gas by collateral ventilation (Scott & Morgan, 2024). All these factors potentially contribute to asymmetry in MIGET reports.

By contrast, since our simplified model does not report asymmetry, effective adjustments of logSD and meanV/Q likely have a smoothing effect, with venous admixture and alveolar dead space partitioning largely unchanged. The logarithmic scale also reduces high V/Q range asymmetry. Clinical evaluation will show whether this approach offers a practical balance between complexity and accuracy in V/Q analysis.

## 5 CONCLUSIONS

Single measurements of arterial blood gases, cardiac output, and indirect calorimetry data, when combined with PaN_2_ measurements, provide sufficient information for deep‐learning‐based applications to quantify individual lung function with diagnostic fidelity in terms of the West V/Q model. The method could be adapted for novel estimates of “true” alveolar dead space (V/Q = ∞). Implementation requires the development of accurate point‐of‐care PaN_2_ measurements across a wide range.

## AUTHOR CONTRIBUTIONS

TJ Morgan developed the project concept. PH Scott devised the Python program, which generated the dataset and implemented the deep‐learning procedures and sensitivity analyses. PH Scott and TJ Morgan wrote Supporting Information File 1 and PH Scott generated the figures. CM Anstey designed and implemented the statistical analysis. All three authors contributed to the writing of the manuscript and reviewed and approved the final version.

## FUNDING INFORMATION

Project supported by Departmental Funds.

## CONFLICT OF INTEREST STATEMENT

The authors declare that they have no competing interests.

## ETHICS STATEMENT

The study did not involve human participants so an ethics statement was not required. The study was performed as a simulation in silico.

## Supporting information


**Data S1:** Supporting Information.

## Data Availability

Expressions of interest for data availability can be directed to the corresponding author.
