# Supplementary material for: Adding arterial nitrogen pressure to single‐measurement monitoring data enables diagnostic lung modeling by deep learning
Source: Physiol Rep. 2026 Feb 16;14(4):e70647. doi: 10.14814/phy2.70647 (PMC12910120; doi:10.14814/phy2.70647)
Supplement: Supplementary file 1 — Data S1: Supporting Information. [file PHY2-14-e70647-s001.docx]

**Supplementary File 1:**

Adding arterial nitrogen pressure to single-measurement monitoring data enables diagnostic lung modelling by deep learning. Physiological Reports.

Peter H. Scott

Intensive Care Department, Mater Health Services, Stanley Str eet, South Brisbane, Brisbane, QLD 4101, and University of Queensland, Brisbane, QLD, 4072, Australia.

ORCID iD: 0000000264286867

Christopher M. Anstey

University of Queensland, Brisbane, QLD, 4072, and Griffith University, Gold Coast, QLD 4215, Australia. ORCID iD: 0000-0003-3636-0107

Thomas J. Morgan

Mater Research and University of Queensland, Stanley Street, South Brisbane, Brisbane, QLD 4101, Australia.

Corresponding author

Peter H. Scott

Intensive Care Department, Mater Health Services, Stanley Street, South Brisbane, Brisbane, QLD 4101, and University of Queensland, Brisbane, QLD, 4072, Australia.

Email: p.scott1@uq.edu.au

The modified West approach.

The modified West approach and the mathematical lung model have been described previously (1, 2). An important insight from studies using the multiple inert gas elimination technique was that the lung units could be modelled with V/Q ratios being log normally distributed. In setting up the V/Q distributions in multiple compartments, West originally used standard deviation values for distributions of compartmental blood flow (Q) and ventilation (V) separately but linked by a mean V/Q value. It was subsequently shown a single value for LogSD being the difference between log standard deviation values for distributions of Q and V could replace those two values. This modified approach renders V of each compartment dependent upon Q and its V/Q ratio. Apart from measurable inputs, the lung model requires three defining parameters being the percentage of total cardiac output not participating in gas exchange (Shunt), the log of the standard deviation of the distribution of V/Q ratios among lung units (LogSD) and the mean V/Q ratio of lung units (MeanV/Q). If these three parameters are known, the lung model, which works by iteratively balancing the uptake of oxygen and excretion of CO_2_ both globally and for each compartment can derive an array of values including partial pressures of gases in mixed venous blood, arterial blood and alveolar gas. By defining the ideal compartment as the one with a R value for blood gas exchange the same as the global R value, West subsequently derived venous admixture using the Shunt equation and derived alveolar dead space (V_DVQ_) using values for ideal alveolar PCO_2_ and mean alveolar PCO_2_ (mPACO_2_). (3) This approach to V_DVQ_ derivation excludes the dead space ventilation of unperfused alveoli defined as V_Dz1_ in the text and analogous to West Zone 1 dead space (4). In clinical practice, the gas evaluated by the technique of volume capnometry represents a mixture of gas from West Zone 1 alveoli and gas from perfused alveoli. Thus, differences between values for mPACO_2_ derived by the lung model and volume capnometry would represent a novel method to enable distinction of West Zone 1 dead space from alveolar dead space due to V/Q mismatch. Knowledge of this distinction may assist in understanding an individual patient`s pulmonary pathology and in titration of positive end expiratory pressure in ventilated patients. LogSD with a range in our study from 0.4 to 2.0 quantifies the spread of V/Q ratios and could be used to quantify the severity of lung injury separate to Shunt and independent of FiO_2_. Nonetheless, the partitioning of venous admixture into Shunt and effective shunt, and the reporting of alveolar dead space may be of greater interest to clinicians than the less familiar concepts of LogSD and MeanVQ.

Alveolar dead space equation (3) :

V_DVQ_/V_T_ = (P_i_ – P_e_)/ P_i_

where: Inspired PCO_2_ = 0

V_DVQ_/V_T_ is the fraction of total alveolar ventilation that is dead space. V_T_

represents ventilation of perfused alveoli.

P_i_ is the alveolar PCO_2_ of the ideal compartment

P_e_ is mPACO_2_

**Fig S1** *Top panels*: Shunt (%) prediction by deep learning on the test dataset as a scatter plot and kernel density estimation. *Bottom-left panel*: Bland-Altman plot of percentage difference for predicted Shunt and true values. *Bottom-right panel*: Scatterplot of Shunt prediction error vs FiO_2_. n = 43915 for all plots except Bland-Altman (n = 500)


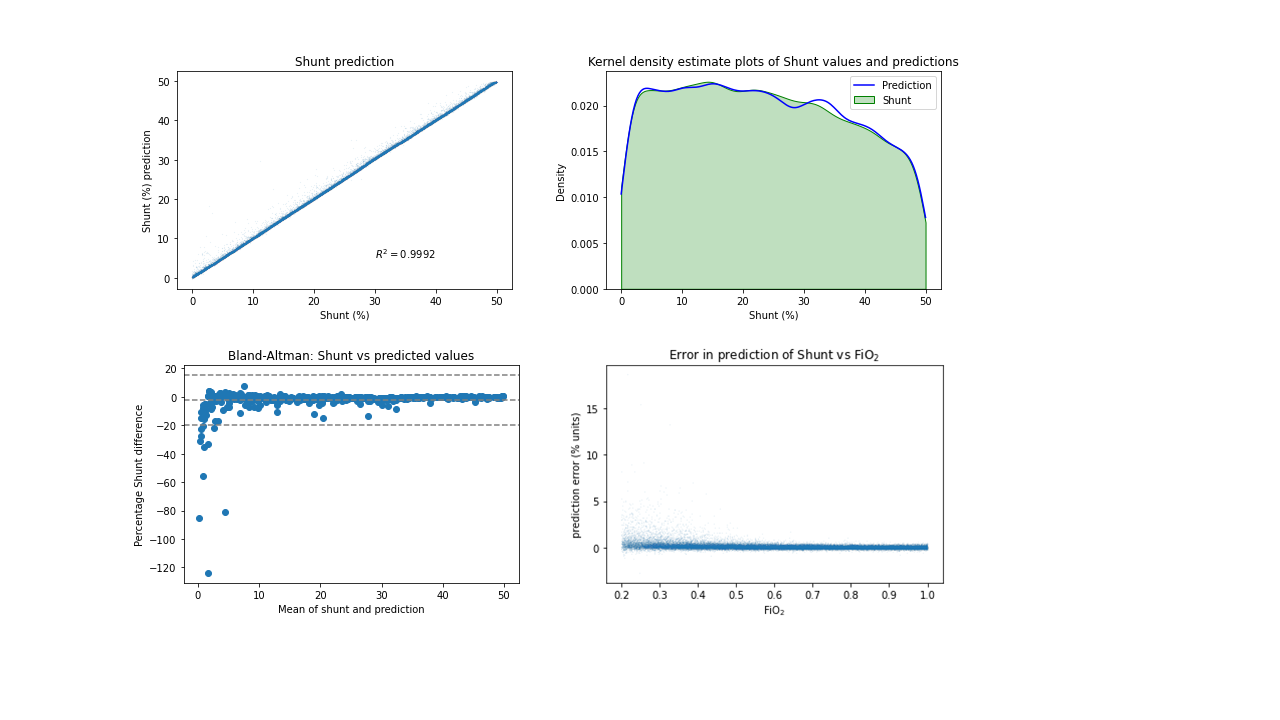


**Fig S2** *Top panels*: LogSD prediction by deep learning on the test dataset as a scatter plot and kernel density estimation. *Bottom-left panel*: Bland-Altmann plot of percentage difference for predicted LogSD and true values. *Bottom-right panel*: Scatterplot of LogSD prediction error vs FiO_2_. n= 43915 for all plots except Bland-Altman (n= 500).


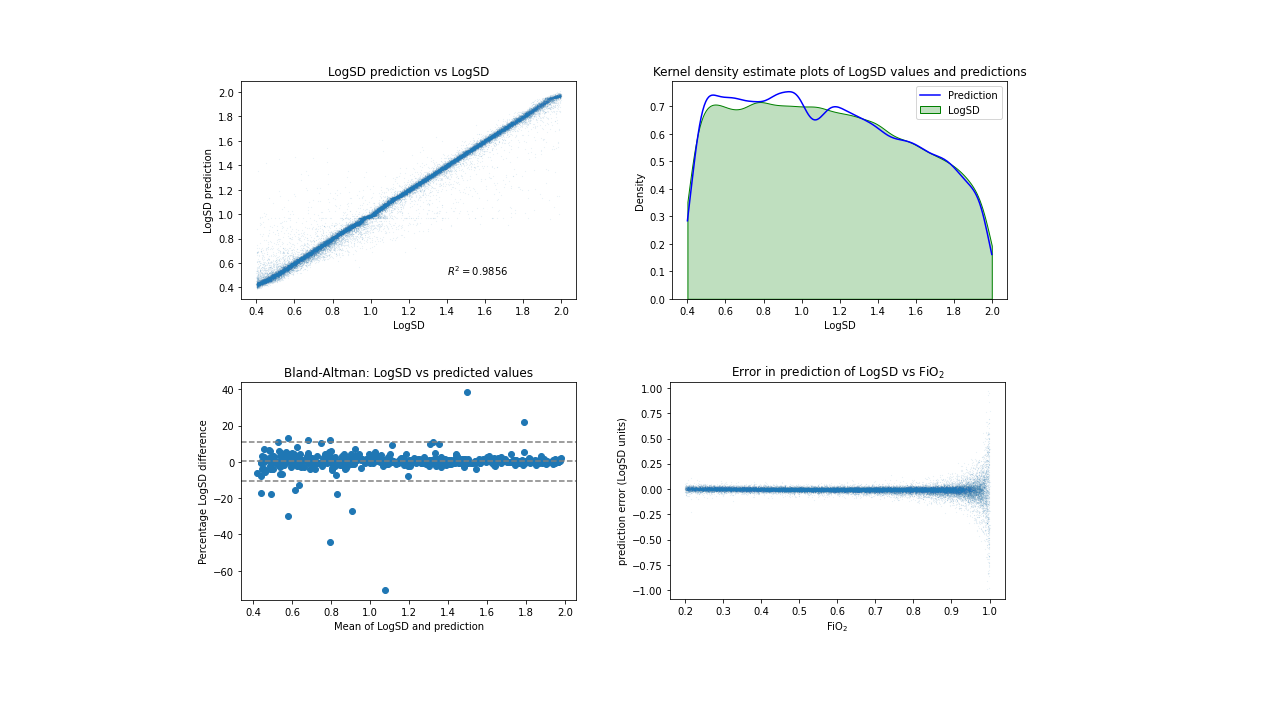


**Fig S3** *Top panels*: MeanV/Q prediction by deep learning on the test dataset as a scatter plot and kernel density estimation. *Bottom-left panel*: Bland-Altman plot of percentage difference for predicted MeanV/Q and true values. *Bottom-right panel*: Scatterplot of MeanV/Q prediction error vs FiO_2_. n= 43915 for all plots except Bland-Altman (n= 500).


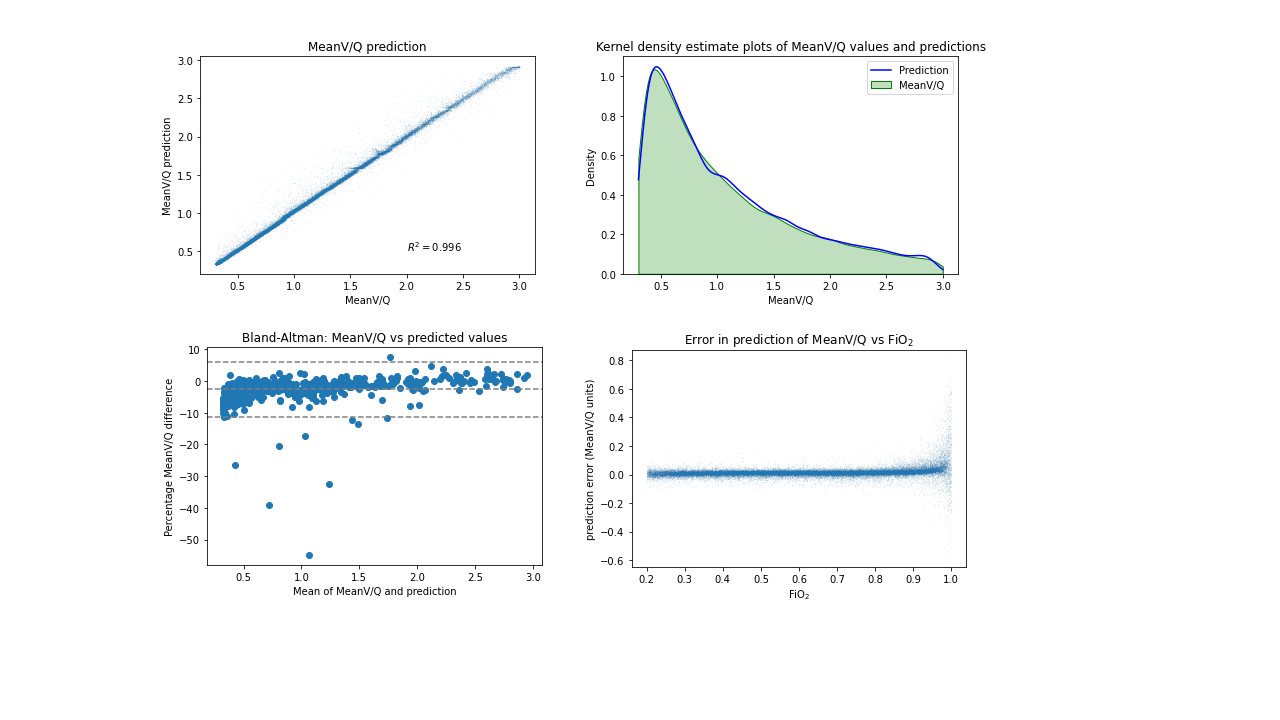


Figure S4 graphs ‘forward’ model calculations of PaN_2_ values from recovered Shunt, LogSD, and MeanV/Q estimates versus ‘true’ PaN_2_ values. Similar plots for PaO_2_, PaCO_2_, arterial pH, and SaO_2_ follow in Figures S5-8. The high reproducibility of variables used as input features confirms the integrity of the underlying mathematical process. Figures S9-11 display plots for ‘forward’ model calculations of mPACO_2_, venous admixture and alveolar dead space using recovered Shunt, LogSD and MeanV/Q estimates versus ‘true’ values.

**Fig S4** Scatterplot of PaN_2_ predictions by forward calculation versus true values for the test dataset. (n = 43915)


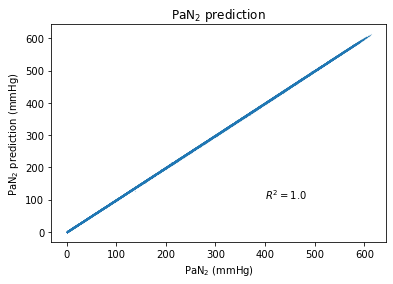


**Fig S5** PaO_2_ prediction by forward calculation vs true values for the test dataset. (n = 43915)


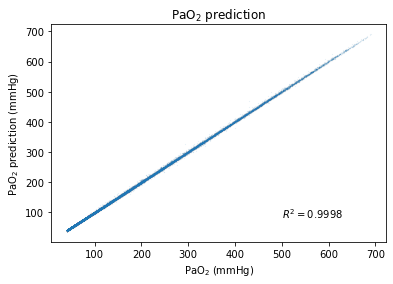


**Fig S6** PaCO_2_ prediction by forward calculation vs true values for the test dataset. (n = 43915)


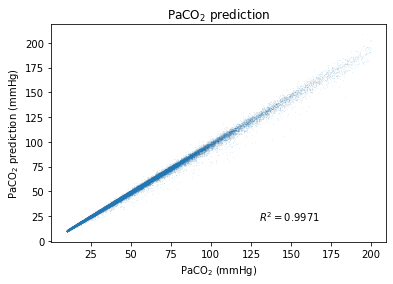


**Fig S7** Arterial pH prediction by forward calculation vs true values for the test dataset. (n = 43915)


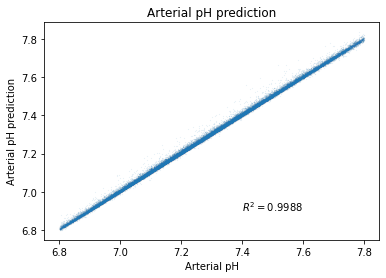


**Fig S8** Arterial O_2_ saturation of haemoglobin (SaO_2_) prediction by forward calculation vs true values for the test dataset. (n = 43915).


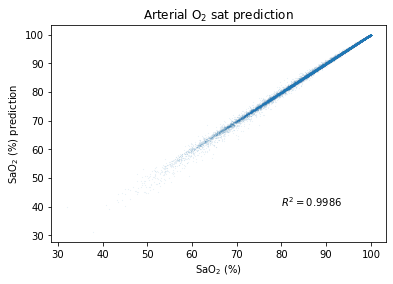


**Fig S9** mPACO_2_ prediction by forward calculation versus true values for the test dataset. (n = 43915)


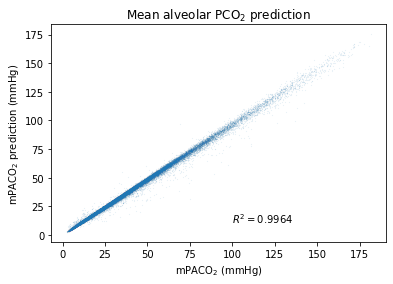


**Fig S10** Venous Admixture prediction by forward calculation vs true values for the test dataset. (n = 43915)


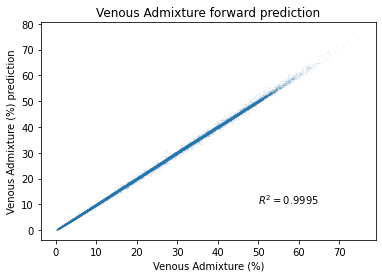


**Fig S11** Alveolar Dead Space prediction by forward calculation vs true values for the test dataset. (n = 43915).


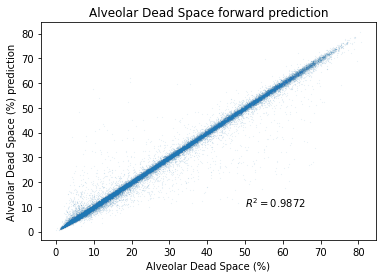


Notes regarding Fig S12-14:

The SHAP sensitivity analysis is designed to identify the weighting the deep learning model places upon respective input features. The approach is not designed to show causality. The issues are clearly shown in relation to the beeswarm plot for LogSD (Fig S13) where FiO_2_ and PaN_2_ have influences upon the determination of LogSD in opposite directions. When FiO_2_ is higher, PaN_2_ will be lower in magnitude. Thus, high FiO_2_ results in a higher LogSD result, but indirectly leads to a lower PaN_2_ value which in turn contributes to a lower LogSD result. The two influences are in balance. This highlights the sensitivity to accurate PaN_2_ measurement, as errors in measurement of PaN_2_ will lead to significant LogSD error prediction.

For the Shunt prediction, high FiO_2_ and resultant high PaO_2_ and SaO_2_ favour a lower value for Shunt. However, high FiO_2_ will be associated with a lower PaN_2_ which favours a higher measurement for Shunt. Thus, the first three influences are balanced against the influence of PaN_2_. The excess of influence is balanced by the other factors.

Thus, while these analyses give much information about sensitivity to error in measurement of an input feature, conclusions regarding physiological causality are not intended.

**Fig S12** SHAP value analysis of input features for the Shunt prediction model. n=500. The SHAP value is the amount by which the input feature has caused the result for Shunt to differ from the average predicted value. Colour coding represents high vs low values of the input feature. PaN_2_ = arterial partial pressure of nitrogen; PaO_2_ = arterial partial pressure of oxygen; PaCO_2_ = arterial partial pressure of CO_2_; VCO_2_ = rate of consumption of CO_2_; SaO_2_ = arterial saturation of haemoglobin with oxygen; FiO_2_ = fractional inspired oxygen; Q_T_ = cardiac output, Hb = Haemoglobin concentration, R = respiratory quotient.


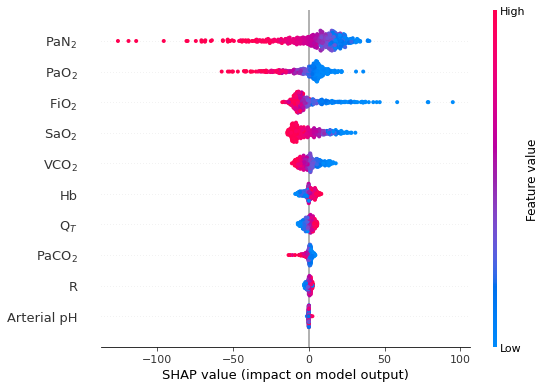


**Fig S13** SHAP value analysis of input features for the LogSD prediction model. n=500. The SHAP value is the amount by which the input feature has caused the result for LogSD to differ from the average predicted value. Colour coding represents high vs low values of the input feature. PaN_2_ = arterial partial pressure of nitrogen; PaO_2_ = arterial partial pressure of oxygen; PaCO_2_ = arterial partial pressure of CO_2_; VCO_2_ = rate of consumption of CO_2_; SaO_2_ = arterial saturation of haemoglobin with oxygen; FiO_2_ = fractional inspired oxygen; Q_T_ = cardiac output, Hb = Haemoglobin concentration, R = respiratory quotient.


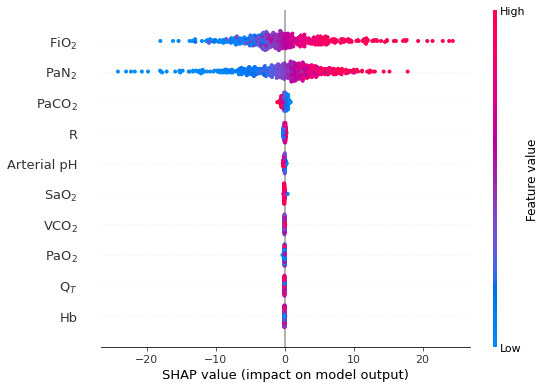


**Fig S14** SHAP value analysis of input features for the MeanV/Q prediction model. n=500. The SHAP value is the amount by which the input feature has caused the result for MeanV/Q to differ from the average predicted value. Colour coding represents high vs low values of the input feature PaN_2_ = arterial partial pressure of nitrogen; PaO_2_ = arterial partial pressure of oxygen; PaCO_2_ = arterial partial pressure of CO_2_; VCO_2_ = rate of consumption of CO_2_; SaO_2_ = arterial saturation of haemoglobin with oxygen; FiO_2_ = fractional inspired oxygen; Q_T_ = cardiac output, Hb = Haemoglobin concentration, R = respiratory quotient.


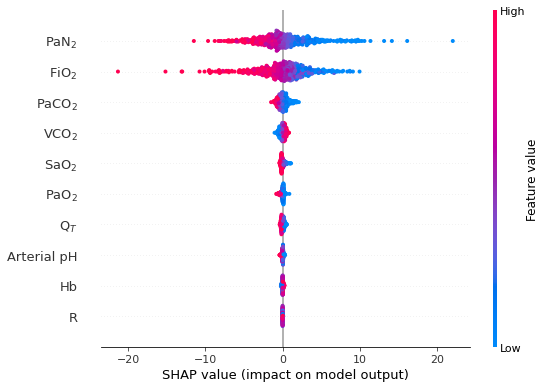


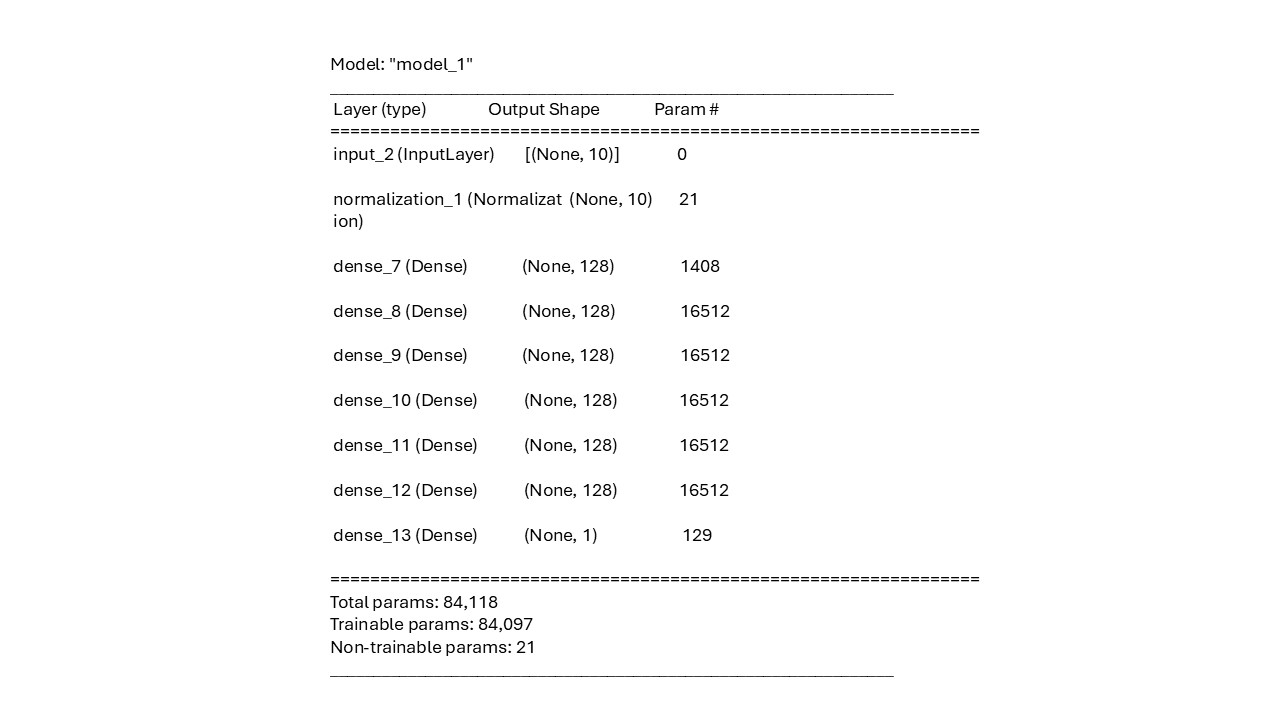


**Table S1** Summary of the model as presented by the Keras package. The first hidden layer is called “dense_7” and the second hidden layer “dense_8” and so on. The output shape “None” substitutes for batch size.


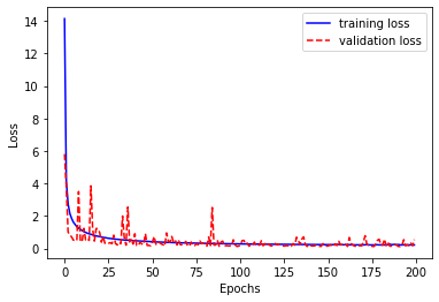


**Fig S14** Training and validation curves using the metric of ‘loss’ for the Shunt prediction model. Similar curves were obtained for the prediction of LogSD and MeanV/Q.


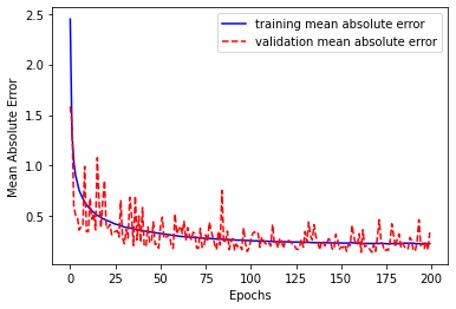


**Fig S15** Training and validation curves using the metric of ‘mean absolute error’ for the Shunt prediction model. Similar curves were obtained for the prediction of LogSD and MeanV/Q.

**Sample solutions**

**Section 1. ‘Single-FiO_2_’ estimates of West lung model parameters by deep learning using measured PaN_2_ values.**

Based on published statistics for patients with ARDS (5-7), we generated a single set of arterial blood gases linked to typical monitoring data which were also fixed (Table S2). FiO_2_ was varied from 0.5 to 0.9 in 0.05 increments, mimicking a progression from moderate to severe ARDS by Berlin criteria (8). PaN_2_ was adjusted at each FiO_2_ step so that model LogSD values calculated by deep learning indicated either normal (~0.45) or marked (~2.0) V/Q heterogeneity. Venous admixture was recorded at each step.

Table S2. Blood gas and monitoring data.

| PaO_2_ | 83 | mm Hg |
| --- | --- | --- |
| PaCO_2_ | 43 | mm Hg |
| SaO_2_ | 0.97 |  |
| Arterial pH | 7.3 |  |
| Hemoglobin | 12 | g/dL |
| VCO_2_ | 211 | mL/min |
| R | 0.92 |  |
| Cardiac Output | 6.0 | L/min |

Using the data inputs in Table S2 and imposed PaN_2_ settings as above, deep-learning estimates of Shunt along with calculated Venous Admixture at each FiO_2_ step are presented in Figures S16 and S17.


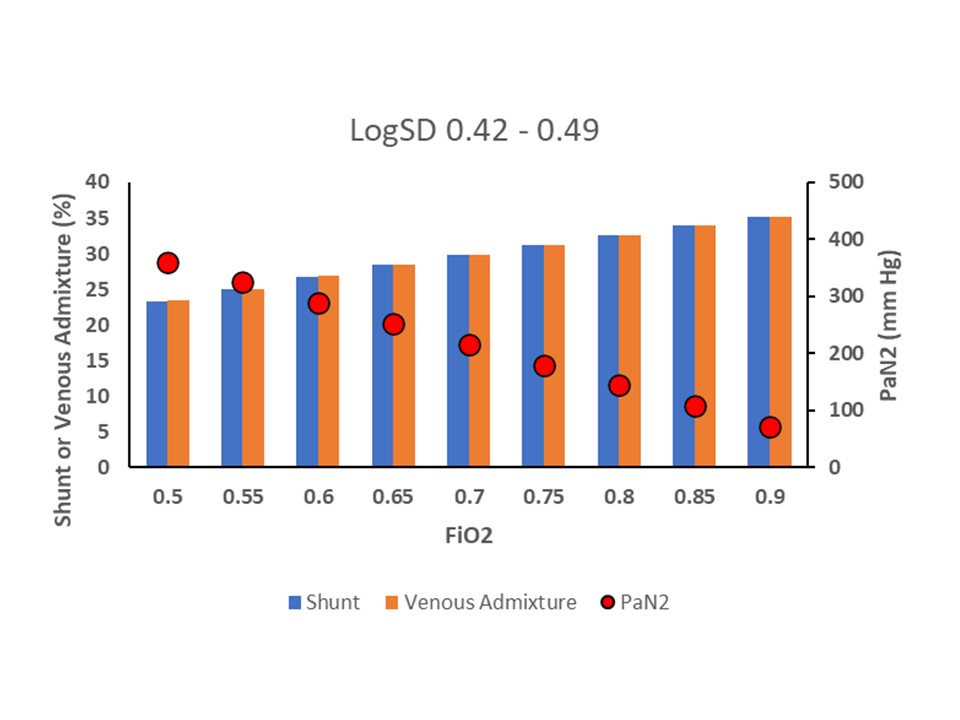


**Figure S16.** Single-FiO_2_ estimates of Shunt at PaN_2_ settings generating LogSD values consistent with normal V/Q heterogeneity, with corresponding venous admixture calculations. MeanV/Q estimates ranged from 0.94 at FiO_2_ 0.5 to 1.15 at FiO_2_ 0.9.


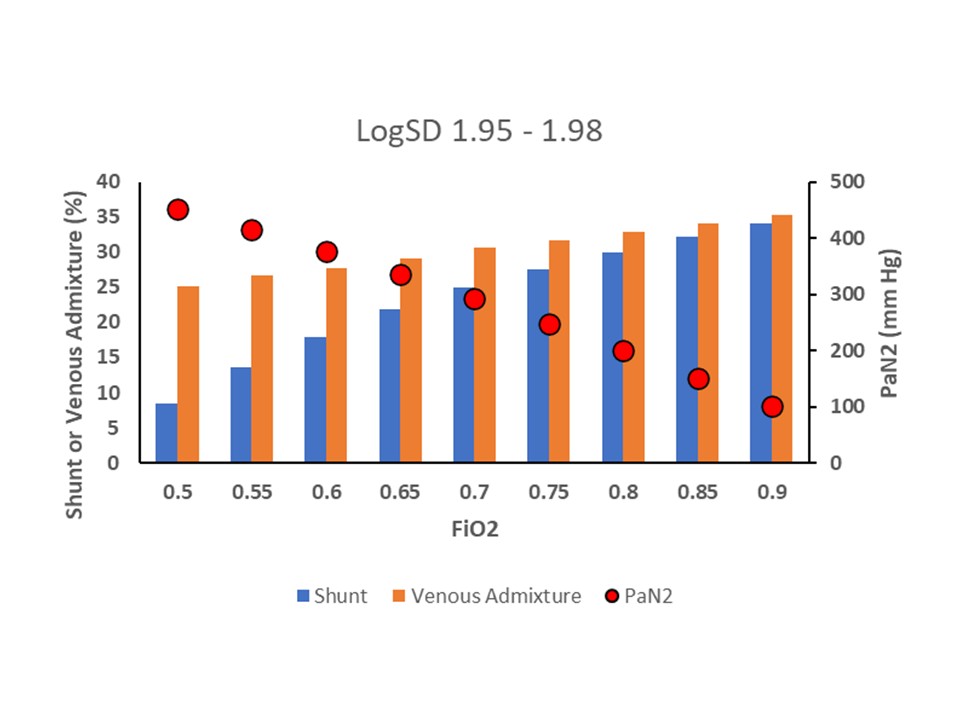


**Figure S17.** Single-FiO_2_ estimates of Shunt at PaN_2_ settings generating LogSD values consistent with increased V/Q heterogeneity, with corresponding venous admixture calculations. MeanV/Q estimates ranged from 0.29 at FiO_2_ 0.5 to 0.48 at FiO_2_ 0.9.

**Comments**

The 18 worked examples used to produce Figures S16 and S17 demonstrate how including measured PaN_2_ values in deep-learning data inputs enables individual ‘Single-FiO_2_’ West parameter solutions for Shunt, LogSD and Mean V/Q.

In Figure S16, LogSD estimates indicated normal V/Q heterogeneity (LogSD ~ 0.45). Under these conditions Shunt accounted for > 99% of venous admixture at all FiO_2_ settings. By contrast, Figure S17 reveals that with significant V/Q heterogeneity (LogSD ~ 2.0), low V/Q unit contributions to venous admixture were considerable at lower FiO_2_ settings. For example, Shunt accounted for just 33% of venous admixture at FiO_2_ 0.5, with the remaining 67% due to low V/Q units. As FiO_2_ rose low V/Q contributions diminished, so that when FiO_2_ reached 0.9 Shunt was responsible for 98% of venous admixture.

In other words, Single-FiO_2_ LogSD assessments incorporating measured PaN_2_ values remain independent quantifiers of V/Q heterogeneity irrespective of the FiO_2_, unlike corresponding low V/Q contributions to venous admixture which are FiO_2_ dependent. Only LogSD determinations allow clinicians to recognize hidden V/Q heterogeneity at higher FiO_2_, when aggressive recruitment manoeuvres and PEEP settings might be inappropriate, as discussed in the main article.

**Section 2: ‘True’ alveolar dead space (V_Dz1_) calculation**

The V_Dz1_ calculation attributes differences between mean alveolar PCO_2_ estimated by deep learning (incorporating PaN_2_ inputs) versus measured values by volumetric capnometry to dilution of alveolar gas with unchanged inspiratory gas from Zone 1 areas. Zone 1 is not included in the West model of pulmonary gas exchange.

Defining V_Dz1_ as the minute volume of ‘true’ alveolar dead space ventilation (V/Q = ∞), the calculation is as follows:

$$V_{Dz1}=\frac{mPACO2(calc)\times VA(calc)}{mPACO2(meas)}-VA(calc)$$

where mPACO_2_(calc) is ‘estimated’ mean alveolar PCO_2_, mPACO_2_(meas) is ‘measured’ mean alveolar PCO_2_ and VA(calc) is estimated total alveolar ventilation.

VA(calc) and mPACO_2_(calc) values are determined by forward calculation of the West model after inserting deep-learning estimates of Shunt, LogSD, and MeanV/Q.

To illustrate the above method of V_Dz1_ calculation, we again adopted Table S2 inputs with a PaN_2_ value of 310 mm Hg and FiO_2_ 0.6. With mPACO_2_(meas) set at 25 mm Hg, V_Dz1_ is calculated to be 1.35 L/min (Table S3).

**Table S3. Results including V_Dz1_ calculation.**

| Shunt | 25.5 | % |
| --- | --- | --- |
| LogSD | 1.13 |  |
| MeanV/Q | 0.7 |  |
| Venous admixture | 26.8 | % |
| VA(calc) | 5.9 | L/min |
| mPACO_2_(calc) | 30.7 | mm Hg |
| mPACO_2_(meas) | 25 | mm Hg |
| V_Dz1_ | 1.35 | L/min |

**References**

1. Morgan TJ, Scott PH, Langley AN, Barrett RDC, Anstey CM. Single-FiO(2) lung modelling with machine learning: a computer simulation incorporating volumetric capnography. J Clin Monit Comput. 2023;37(5):1303-11.

2. Scott PH, Morgan TJ. Multi-compartment V/Q lung modeling: Log normal distributions of inspired or expired alveolar gas? Physiol Rep. 2024;12(17):e16175.

3. West JW, PD. . Pulmonary gas exchange. In: JB W, editor. Bioengineering Aspects of the Lung. New York: Marcel Dekker; 1977. p. 361-457.

4. West JB, Dollery CT, Naimark A. Distribution of blood flow in isolated lung; relation to vascular and alveolar pressures. J Appl Physiol. 1964;19:713-24.

5. Sayed M, Riano D, Villar J. Novel criteria to classify ARDS severity using a machine learning approach. Crit Care. 2021;25(1):150.

6. Wang Z, Zhang L, Huang T, Yang R, Cheng H, Wang H, et al. Developing an explainable machine learning model to predict the mechanical ventilation duration of patients with ARDS in intensive care units. Heart Lung. 2023;58:74-81.

7. Karbing DS, Panigada M, Bottino N, Spinelli E, Protti A, Rees SE, Gattinoni L. Changes in shunt, ventilation/perfusion mismatch, and lung aeration with PEEP in patients with ARDS: a prospective single-arm interventional study. Crit Care. 2020;24(1):111.

8. Force ADT, Ranieri VM, Rubenfeld GD, Thompson BT, Ferguson ND, Caldwell E, et al. Acute respiratory distress syndrome: the Berlin Definition. JAMA. 2012;307(23):2526-33.
